# Molecular diagnosis of autosomal dominant congenital cataract in two families from North India reveals a novel and a known variant in *GJA8* and *GJA3*

**DOI:** 10.3389/fped.2022.1003909

**Published:** 2022-12-02

**Authors:** Vanita Vanita, Shiwali Goyal, Shailja Tibrewal, Suma Ganesh

**Affiliations:** ^1^ Department of Human Genetics, Guru Nanak Dev University, Amritsar, India; ^2^ Department of Pediatric Ophthalmology, Strabismus and Neuro-Ophthalmology, Dr. Shroff's Charity Eye Hospital, Daryaganj, India; ^3^ Department of Ocular Genetics, Dr. Shroff’s Charity Eye Hospital, Daryaganj, India

**Keywords:** ADCC, crystallins, *GJA3*, *GJA8*, nuclear cataract, total cataract, sequencing

## Abstract

**Aims:**

The study aims to detect the underlying genetic defect in two autosomal dominant congenital cataract (ADCC) families.

**Methods:**

A detailed family history was collected, pedigrees were drawn, and slit-lamp examination and lens photography were performed. Mutation screening was carried out in the genes for crystallins and connexins by PCR and Sanger sequencing. Ethnically matched controls were tested for the identified variants. Different bioinformatics tools were used to assess the pathogenicity of the observed variants.

**Results:**

In an ADCC family with total cataract, a novel change (c.166A > G) (p.Thr56Ala) in *GJA8* was identified. In another ADCC family with nuclear cataract, c.134G > C (p.Trp45Ser) in *GJA3* has been detected. These variants co-segregated completely in patients in their respective families and were neither observed in unaffected family members nor in ethnically matched 100 controls, excluding them as polymorphisms.

**Conclusions:**

The present study identifies a novel variant c.166A > G (p.Thr56Ala) in *GJA8* in an ADCC family having total cataract and a previously known mutation c.134G > C (p.Trp45Ser) in *GJA3* in another ADCC family. Thr56 in *GJA8* seems to be a mutation hotspot, as previously an ADCC Mauritanian family harbored a different substitution (p.Thr56Pro) at the same codon, although for a different phenotype (nuclear cataract). Similarly, Trp45 in *GJA3* appears as a mutation hotspot, as p.Trp45Ser has previously been reported for nuclear cataract in a Chinese ADCC family. p.Thr56 (GJA8) and p.Trp45 (GJA3*)* are in the extracellular loop 1 (EL1) in their respective connexin proteins, which, along with EL2, are essential for gap junction formation, hemichannel docking, and regulating the voltage gating of the channels. Hence, residues in these regions seem crucial for maintaining eye lens transparency.

## Introduction

Congenital cataract, an opacification of the eye lens present at birth or early in the postnatal period, is the primary cause of visual impairment in children, with an incidence ranging from 0.12 to 13.6 per 10,000 live births (1). Congenital cataracts cause 5%–20% of childhood blindness in developed countries and 22%–30% in developing countries (2). Congenital cataract can occur either as an isolated eye anomaly, in association with other ocular anomalies, or as a component of multisystemic disorders. Approximately 33% of the cases have a positive family history and predominantly follow the autosomal dominant mode of inheritance (3). Extensive clinical (with various types and subtypes) and genetic heterogeneity (>50 loci and mutations in >35 genes at these loci for the nonsyndromic type) have been observed for congenital cataract (https://cat-map.wustl.edu).

Crystallins are the major structural and functional lens proteins that are categorized into three groups: alpha [*CRYαA* (OMIM 123580) and *CRYαB* (OMIM 123590)]; beta [*CRYβA1/A3* (OMIM 123610), *CRYβA2* (OMIM 600836), *CRYβA4* (OMIM 123631), *CRYβB1* (OMIM 600929), *CRYβB2* (OMIM 123620), and *CRYβB3* (OMIM 123630)]; and gamma [*CRYγA* (OMIM 123660), *CRYγB* (OMIM 123670), *CRYγC* (OMIM 123680), *CRYγD* (OMIM 123690), and *CRYγS* (OMIM 123730)], which constitute up to 90% of the total soluble lens proteins and their stability and proper interactions are critical for lens transparency. The eye lens is an avascular organ, and intercellular transport of small biomolecules (≤1 kDa) (cyclic-AMP, K^+^, glucose, inositol triphosphate) is mediated through connexins (Cx), also known as gap junction channel proteins. Connexins are encoded by at least 21 genes in humans, and these are classified into five groups: [*α* (*GJA1*, *GJA3*, *GJA4*, *GJA5*, *GJA8*, *GJA9*, and *GJA10*), *β* (*GJB1*, *GJB2*, *GJB3*, *GJB4*, *GJB5*, *GJB6*, and *GJB7*), *γ* (*GJC1*, *GJC2*, and *GJC3*), *δ* (*GJD2*, *GJD3*, and *GJD4*), and *ɛ* (*GJE1*)] (4). Connexin 43 (Cx43, *GJA1*), connexin 46 (Cx46, *GJA3*), and connexin 50 (Cx50, *GJA8*) belonging to the *α*-connexin gene family, are expressed in the human lens. Cx43 and Cx50 are the major connexins in the epithelial cells. Diverse gap junction channels formed by Cx43 and Cx50 subunits are important for the differentiation, elongation, and maturation of the lens fiber cells. During differentiation, Cx43 expression is reported to be downregulated and Cx46 is highly expressed, while mature lens fiber cells express both Cx46 and Cx50 (5–7). Cx46 and Cx50 are instrumental in joining the lens cells into a functional syncytium (8, 9). In addition, Cx46 is essential for lens transparency, maintaining Ca^2+^ homeostasis in the nucleus, and Cx50 is required for cell proliferation, differentiation, transparency, and lens growth (10). Lens fibers are connected to lens epithelial cells *via* gap junctions and are dependent on a metabolically active epithelium for the maintenance of the intracellular ionic conditions that are necessary to prevent precipitation of crystallins and, hence, cataract formation (11). In congenital cataract families with identified causative genes, nearly 37% have been linked with mutations in crystallins, about 22% in connexins, and approximately 14% in transcription factors, and the remaining cases are documented to harbor mutations in a variety of other genes (12).

The present study intended to detect the underlying genetic defects in two autosomal dominant congenital cataract (ADCC) families (PC-04 and PC-12) with total and nuclear cataract, respectively, encountered at the Dr. Shroff's Charity Eye Hospital (Dr. SCEH), Daryaganj, New Delhi, India. Upon sequence analysis of the genes for crystallins (*CRYαA*, *CRYαB*, *CRYβA1/A3*, *CRYβA2*, *CRYβA4*, *CRYβB1*, *CRYβB2*, *CRYβB3*, *CRYγA*, *CRYγB*, *CRYγC*, *CRYγD*, and *CRYγS*) and connexins [*GJA3* (OMIM 121015) and (*GJA8* (OMIM 600897)] in affected members of the PC-04 family, we have identified a previously unreported heterozygous c.169A > G change in *GJA8* that resulted in the substitution of a highly conserved threonine by alanine at codon 56 (p.Thr56Ala). Affected members of another ADCC family, PC-12, showed c.134G > C substitution in *GJA3* that resulted in the replacement of highly conserved tryptophan by serine at codon 45 (p.Trp45Ser), a previously known change for bilateral congenital cataract in a Chinese ADCC family. p.Thr56Ala and p.Trp45Ser changes co-segregated completely with the disease phenotypes in the respective ADCC families.

## Material and methods

### Family description

The present study is a hospital-based study. Two congenital cataract families were recruited from the Dr. SCEH, where patients affected by different eye diseases, belonging to different parts of India, visit for clinical checkups and surgeries. This study was approved by the Ethics Review Board of the Guru Nanak Dev University (GNDU) and the Dr. SCEH, in accordance with the provisions of the Declaration of Helsinki. Informed consent was obtained from each adult (and the parents of minors) studied. Detailed—three- to four-generation family history was recorded according to the nomenclature provided by Bennett et al. (13) followed by a slit-lamp examination and lens photography.

**Figure 1:**
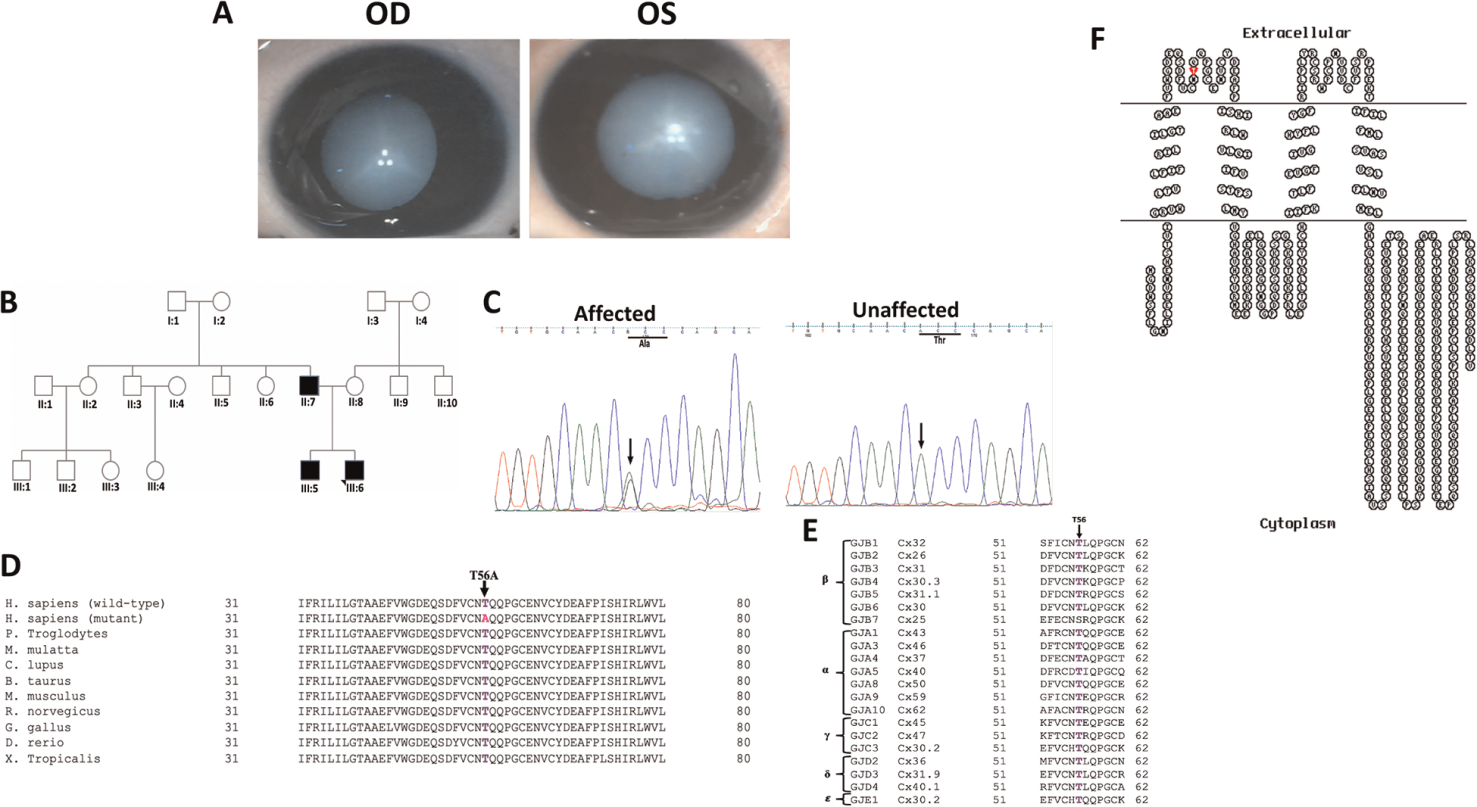
(**A**) Clinical photographs (OD and OS) of the proband (III:6) of the PC-04 family indicating total cataract. (**B**) Pedigree of an autosomal dominant congenital cataract family indicating three affected individuals in two generations. The proband is indicated with an arrow. Squares and circles symbolize male and female individuals, respectively. (**C**) Electropherograms of a part of the reverse strand sequence of exon 2 of *GJA8* in the affected individual (III:6). Arrow indicates the nucleotide at which heterozygous change (AG) occurred that resulted in c.166A > G (p.T56A) in all the affected individuals. Also shown is a part of reverse strand sequence of exon 2 of *GJA8* in an unaffected individual (II:8) indicates wild-type (AA) genotype. (**D**) Multiple amino acid sequence alignment (NCBI HomoloGene) of *GJA8* indicating conservation of threonine [T] (shown in purple) at position 56 as indicated by an arrow in different species. Mutant sequence [*Homo sapiens* (mutant)] (replacement of threonine [T] by alanine [A] at 56 position) in the affected members of the present analyzed PC-04 family is highlighted in pink. (**E**) Threonine at position 56 (indicated by an arrow) is also highly conserved in the members of different groups of human gap junction proteins (connexins) except in one of the *β* group connexin, that is, *GJB7*. (**F**) The membrane topological structure of *GJA8* was generated by TOPO2 software (http://www.sacs.ucsf.edu/TOPO2/). The mutation p.T56A (indicated by red dot) is located in the first extracellular loop.

The index case (III:6) in family PC-04, a 15-day-old male child, was diagnosed with bilateral congenital cataract on ophthalmic examination at the Dr. SCEH. He was operated for the total cataract (Figure 1A) in both the eyes (B/E) when he was 3 and 4 month-old. The family history revealed his elder brother (III:5) (a 5-year-old child at the time of presentation) and father (II:7) were both affected with bilateral congenital cataracts (Figure 1B) since birth and both got operated on (B/E) at the age of 5 years, while his mother (II:8) was diagnosed as unaffected. I:1 and I:2 (grandparents of the proband) were not available to participate in the present study.

**Figure 2:**
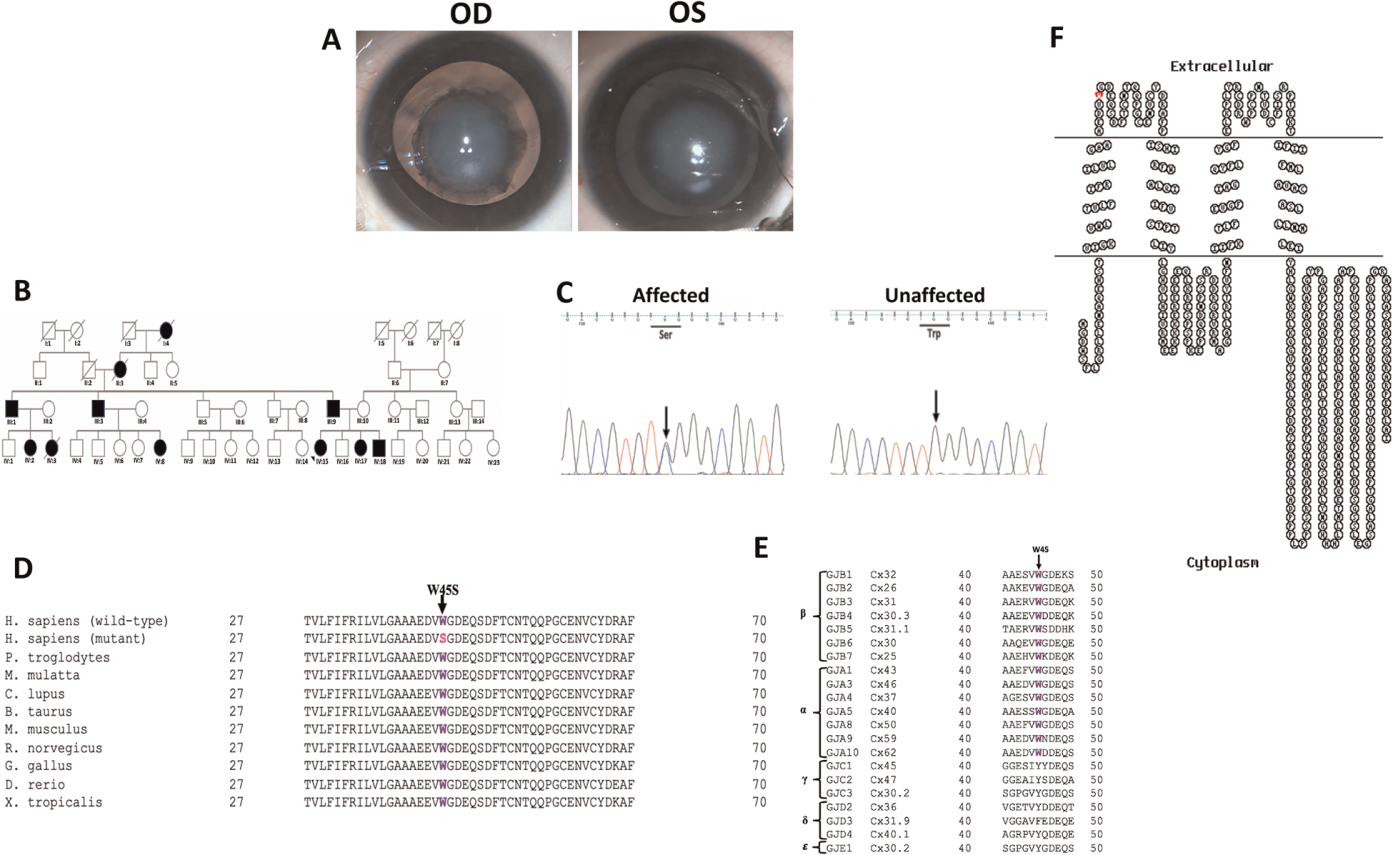
(**A**) Clinical photographs (OD and OS) of the proband (IV:15) of the PC-12 family indicating nuclear cataract. (**B**) Pedigree of an autosomal dominant congenital cataract family indicating 11 members in four generations affected with congenital cataract. The proband is indicated with an arrow. Squares and circles symbolize male and female individuals, respectively. Squares/circles with diagonal lines represent deceased persons. (**C**) Electropherograms of a part of the reverse strand sequence of exon 2 of *GJA3* in the affected individual (IV:15). Arrow indicates the nucleotide at which heterozygous change (GC) occurred that resulted in c.134G > C (p.W45S) in the affected individuals. Also shown is a part of reverse strand sequence of exon 2 of *GJA3* in an unaffected individual (III:10) indicating wild-type (GG) genotype. (**D**) Multiple amino acid sequence alignment (NCBI HomoloGene) of *GJA3* indicating conservation of tryptophan [W] (shown in purple) at position 45 as indicated by an arrow in different species. Mutant sequence [*Homo sapiens* (mutant)] (replacement of tryptophan [W] by serine [S] at 45 position) in the affected members of the present analyzed PC-12 family is highlighted in pink. (**E**) Tryptophan at position 45 (indicated by an arrow) is highly conserved in different members of *β* and *α* human gap junction proteins (connexins). However, it is not conserved in the members of *γ*, *δ*, and *ɛ* connexins, as only subfamily *α* and *β* are sister groups with maximum likelihood of 95% and Bayesian posterior probability of 1 (38). (**F**) The membrane topological structure of *GJA3* was generated by TOPO2 software (http://www.sacs.ucsf.edu/TOPO2/). The mutation p.W45S (indicated by red dot) is in the first extracellular loop.

In the family PC-12, the proband (IV:15) was a 7-year-old female who was diagnosed with the bilateral congenital cataract of nuclear type (Figure 2A). Detailed family history revealed a total of 11 members in four generations (Figure 2B) including her father (III:9), younger sister (4 years old, IV:17), and younger brother (3 years old, IV:18) to be bilaterally affected with congenital cataract.

Three affected (II:7, III:5, and III:6) and an unaffected individual (II:8) in family PC-04 and four affected (III:9, IV:15, IV:17, and IV:18) and two unaffected individuals (III:10 and IV:16) in family PC-12 underwent ophthalmic examination and gave blood samples for the present genetic study. A blood sample of 5–10 ml (intravenously) was collected from each participant in a 15 ml centrifuge tube containing 0.5 M EDTA and genomic DNA isolated using organic solvents (14).

### Mutation screening

Mutation screening was performed in the exonic regions and exon–intron boundaries of the candidate genes: *CRYαA*, *CRYαB*, *CRYβA1/A3*, *CRYβA2*, *CRYβA4*, *CRYβB1*, *CRYβB2*, *CRYβB3*, *CRYγA*, *CRYγB*, *CRYγC*, *CRYγD*, *CRYγS*, *GJA3*, and *GJA8*, using exon specific primers, designed with the help of the Primer select program of the Lasergene package (DNASTAR Inc., Madison, WI, United States) (primer sequences are available in the Supplementary Table S1). Initially, genomic DNA samples from an affected and an unaffected individual from each of these two families were amplified using approximately 50 ng of genomic DNA, 10 pmol of each forward and reverse primers, 200 μM dNTP mix (Bangalore Genei), 10× Taq buffer with 15 mM MgCl_2_ (Bangalore Genei), and 3 U/μl Taq DNA Polymerase (Bangalore Genei) in a 15 μl final reaction volume. A touch-down PCR with the following cycling conditions was performed; initial denaturation at 95°C for 5 min, followed by 2 cycles each of 1 min at 95°C, 1 min at 61°C, 1 min at 72°C; 2 cycles each of 1 min at 95°C, 1 min at 59°C, 1 min at 72°C; 2 cycles each of 1 min at 95°C, 1 min at 57°C, 1 min at 72°C, then 31 cycles each of 1 min at 95°C, 1 min at 55°C, 1 min at 72 °C, and finally an extension step for 10 min at 72 °C. The amplified PCR products were checked on 2%–2.5% agarose gel depending on the size of the amplicons and purified using the QIAquick PCR purification kit (Catalog number 28104). Purified PCR products were sequenced bidirectionally with the BigDye™ Terminator Cycle Sequencing Kit ver. 3.1 (ABI, Foster City, CA, United States) for a 10 μl final volume, containing 2 μl purified PCR product, 1 μl BigDye Terminator reaction mix, 2 μl 5× BigDye sequencing buffer (ABI, Foster City, CA, United States), 1 μl 5 pmol primer (forward and reverse primer in two separate tubes), and 4 μl double-distilled water. Cycling conditions were 96°C for 2 min, 27 cycles at 96°C for 10 s, 55°C for 15 s, and 60°C for 4 min. The sequencing reaction products were purified by the 75% isopropanol precipitation method (ABI protocol), resuspended in 10 μl of Hi-Di formamide (ABI, Foster City, CA, United States), denatured at 95°C for 5 min, and electrophoresed on a 3500xL Genetic Analyzer (Applied Biosystems, Thermo Fisher Scientific). Sequences were assembled and analyzed using the SeqMan II program of the Lasergene package (DNASTAR Inc., Madison, WI, United States). Upon identification of nucleotide substitution in *GJA8* (proband III:6; PC-04 family) and in the *GJA3* (proband IV:15; PC-12 family), other available affected and unaffected individuals were tested for the observed variants in respective families. Furthermore, 100 ethnically matched controls (free from any eye anomaly) were tested for the observed variants in *GJA8* (PC-04 family) and *GJA3* (PC-12 family), which co-segregated completely with the phenotypes to exclude their possibility as polymorphisms in the same population.

### *In silico* analysis

Different *in silico* bioinformatics tools were used to predict the effects of identified missense mutations on the encoded protein. The effect of identified missense variants [*GJA8* c.166A > G, p.Thr56Ala (PC-04 family) and *GJA3* c.134G > C, p.Trp45Ser (PC-12 family)] were interpreted using HOPE software (https://www.cmbi.umcn.nl/hope) and VarCards (http://var-cards.biols.ac.cn/). Using nucleotide change (GJA8:c.166A > G and GJA3:c.134G > C) as an input, aggregated pathogenicity scores (output) were evaluated by VarCards (keeping the default settings), which is based on different algorithms (SIFT, Polyphen-2_HDIV, Polyphen-2_HVAR, LRT, MutationTaster, MutationAssessor, FATHMM, PROVEAN, VEST3, MetaSVM, MetaLR, M-CAP, CADD, DANN, FATHMM_MKL, Eigen, GenoCanyon, fitCons, GERP++, PhyloP, phastCons, SiPhy, REVEL, ReVe, and ClinPred). These algorithms are in accordance with the American College of Medical Genetics (ACMG) standards and are sourced from the dbNSFP v3.0 database (15). The output score values were critically analyzed, and the variants were categorized as damaging/disease causing/pathogenic/conserved, accordingly, based on the cut-off scores (Supplementary Tables S2, S3). For HOPE software, for the input purposes, amino acid change was used (Thr56Ala and Trp45Ser) keeping the default settings, and the output file, which usually shows the hydrophobicity and size difference between the wild-type and mutant proteins and the conservation status for the wild-type amino acid, was analyzed. Additionally, conservation analyses of the amino acids at the altered sites were determined in different species and different human connexins using HomoloGene NCBI (https://www.ncbi.nlm.nih.gov/homologene). Multiple sequence alignment analyses are largely based on the assumption that evolutionarily conserved amino acids are more likely to be functionally important as compared with nonconserved amino acids. Transmembrane protein display was carried out using the TOPO2 software, following the instructions given on the TOPO2 website (http://www.sacs.ucsf.edu/TOPO2/).

## Results

### Phenotype description

The proband (III:6) in family PC-04 had a bilateral whitish reflex in the pupillary area noted by his parents when he was 8 days old. He had an uneventful birth history and no systemic abnormalities. On ophthalmic examination, he was diagnosed with total cataracts in both the eyes. The cataract was dense (Figure 1A) (intraoperative lens photographs taken) and involved the entire lens (the size of the opacities could not be determined), causing obscuration of the visual axis in both the eyes. There were denser opacities along the Y-sutures. The lens was of normal shape and size. There was no microcornea or any other anterior segment abnormalities. There was no nystagmus or strabismus at the time of the presentation. He underwent cataract removal in both the eyes before 4 months of age and secondary intraocular lens implantation at 6 years of age. His best-corrected visual acuity (BCVA) was recorded as 6/18 and 6/9 in the right and left eye, respectively, at his last visit, suggesting right eye deprivation amblyopia. There was latent nystagmus in both the eyes, no strabismus, no glaucoma, and normal retinae and optic nerves. His 25-year-old father (II:7) had a history of bilateral childhood cataract (detected during the first year of his life) and subsequently underwent surgery. He was aphakic with BCVA of 6/24 and 6/60 in the right and left eye, respectively. He had bilateral jerky nystagmus and left-eye esotropia. The proband's elder brother (III:5) was also diagnosed with a bilateral childhood cataract at the age of 5 years and was subsequently operated on. His postoperative BCVA was 6/9 and 6/6 in the right and left eye, respectively.

In the PC-12 family, the proband, a 7-year-old female (IV:15), presented with complaints of blurring of vision in both the eyes since birth as noticed by her parents. The vision in both the eyes was counting fingers close to the face. The cataract was diagnosed as a congenital nuclear type. It was a bilaterally symmetrical, round, central (6 mm in diameter in both eyes), moderately dense opacity involving the embryonic and fetal nucleus (Figure 2A) (intraoperative lens photographs taken). There was no evidence of posterior polar, posterior subcapsular, or anterior subcapsular opacities. The proband had bilateral horizontal jerky nystagmus, and strabismus (exotropia) in the right eye. The 4-year-old younger sister (IV:17) of the proband also showed identical involvement of the lens, nystagmus, similar presenting visual acuity, and left-eye strabismus (esotropia). None of them had microcornea, glaucoma, retinal pathology, or optic nerve disease. Cataract surgery was performed soon after the presentation in both the affected sisters, and their father was also operated bilaterally for congenital cataract in his childhood. In the proband, cataract extraction and intraocular lens implantation recovered the vision to 6/18 in both the eyes. She had bilateral deprivation amblyopia. In her affected sister (IV:17), vision was restored to 6/18 in the right and 6/36 in the left eye after cataract surgery and intraocular lens implantation. She also had bilateral deprivation amblyopia and additional strabismic amblyopia in the left eye. Both the siblings underwent strabismus surgery 1.5 years after cataract surgery.

### Mutation screening

In PC-04 family, bidirectional sequence analysis of the coding regions and the exon–intron boundaries of the candidate genes *CRYαA*, *CRYαB*, *CRYβA1/A3*, *CRYβA2*, *CRYβA4*, *CRYβB1*, *CRYβB2*, *CRYβB3*, *CRYγA*, *CRYγB*, *CRYγC*, *CRYγD*, *CRYγS,* *GJA3,* and *GJA8* showed a previously unreported change in *GJA8*, that is, A > G at nucleotide position 166 (c.166A > G) (Figure 1C) in heterozygous (AG) form in the proband (III:6), which on further analysis was observed in his affected sibling (III:5) and affected father (II:7) as well. The alteration was seen neither in the unaffected mother (II:8) of the proband (wild-type AA homozygous) nor in the 100 unrelated controls (free from any eye anomaly) from the same ethnicity, hence excluding this change as a polymorphism. The c.166A > G substitution replaced threonine with alanine (Figure 1D) at amino acid position 56 (p.Thr56Ala), which is evolutionarily highly conserved in different species and different human connexins (*α*, *β*, *γ*, *δ*, and *ɛ*) (Figure 1E) and is in the extracellular loop 1 (EL1) (Figure 1F) of connexin 50.

Similarly, in the PC-12 family, bidirectional sequence analysis of the coding regions and the exon–intron boundaries of the above-mentioned candidate genes in the proband (IV:15) indicated a c.134G > C change in the heterozygous form (GC) in *GJA3* (Figure 2C), which was further seen in three other available affected members (III:9, IV:17, and IV:18). The alteration was neither seen in the unaffected mother (III:10) and an unaffected sibling (IV:16) of the proband nor in the 100 ethically matched unrelated controls, thus excluding it as polymorphism in the tested Indian population. The c.134G > C substitution replaced tryptophan with serine at amino acid position 45 (p.Trp45Ser), which is evolutionarily highly conserved in different species (Figure 2D) and in different members of human *α*, *β* connexins (Figure 2E), and is in the EL1 (Figure 2F) of connexin 46.

Furthermore, different bioinformatics tools predicted these two changes, c.166A > G in *GJA8* (in PC-04 family) and c.134G > C in *GJA3* (in PC-12 family), to be pathogenic. In our results, scores obtained using different algorithms predicted c.166A > G (p.Thr56Ala) in *GJA8*, to be damaging/disease causing/pathogenic (Supplementary Table S2). The c.166A > G (p.Thr56Ala) variant observed in *GJA8* was not reported in the 1000 genomes (http://www.1000genomes.org/), gnomAD (https://gnomad.broadinstitute.org/), or in the dbSNP databases, indicating this to be a novel variant. Similarly, various functional prediction programs in VarCards showed c.134G > C (p.Trp45Ser) in *GJA3* to be damaging/disease causing/pathogenic (Supplementary Table S3).

## Discussion

In the present study, we report a novel substitution c.166A > G (p.Thr56Ala) in the connexin 50 in an ADCC family with three affected members in two generations having total cataract (PC-04). In another ADCC family having 11 affected members in four generations (PC-12) with nuclear cataract, we have observed c.134G > C (p.Trp45Ser) in *GJA3*, a previously known substitution for ADCC. These substitutions segregated completely with the disease in the affected members in the respective families and were not seen either in the tested unaffected family members or in the 100 unrelated controls tested for each substitution, hence excluding these as polymorphisms.

Cx46 and Cx50 contain four highly ordered transmembrane domains (TM1–TM4) connected by two extracellular loops (EL1–EL2) and a cytoplasmic loop (CL). Transmembrane domains (TM1–TM4) perform a variety of functions, such as enzyme catalysis, transport across membranes, transducing signals as receptors of hormones and growth factors, and energy transfer in ATP synthesis. Extracellular loops (EL1–EL2), the most conserved domains among connexins, are reported to be involved in the hemichannel docking interactions formed between different connexons and can establish homotypic and heterotypic channels (16–18). The six connexins oligomerize in the endoplasmic reticulum to form a connexin hemichannel (also known as a connexon), which traffics to the plasma membrane of one cell where it can dock with another hemichannel from an adjacent cell to form a complete gap junction channel (19, 20). Connexons can be either heteromeric (made up of six distinct connexin protomers) or homomeric (formed by two or more different connexin isoforms). Therefore, docked connexons can result in one of four types of structures: homomeric–homotypic, heteromeric–homotypic, homomeric–heterotypic, or heteromeric–heterotypic (20). EL2 is also assumed to play a role in the selection of pairing partners of lens connexons and controls the activity and permeability properties of the channels (20–23). In the EL1 and EL2 domains, the docking hydrogen bond (HB)-forming residues are concentrated within a relatively small region of only four residues (54–57 in EL1; 168, 176, 177, and 179 in EL2) assisting in the docking HB formation in the Cx26 (20). As EL1 and EL2 are the most conserved domains among connexins, residues in other connexins equivalent to the Cx26 docking HB-forming residues are all assumed to be capable of forming similar HBs to support docking function (20). The NH_2_ and COOH terminal ends of the connexin polypeptides are in the cytoplasm. N-terminal end along with EL1 and TM1/TM2 form the permeation pathway for a variety of cytosolic substrates. Thousands of these channels are clustered in the plane of the plasma membrane to form gap junction plaques. Diverse gap junction channels formed by connexin 46 and connexin 50 subunits are important for differentiation, elongation, and maturation of lens fiber cells (24).

So far, over 50 mutations in *GJA3* and more than 70 mutations in *GJA8* have been documented for different cataract phenotypes in families from different ethnicities worldwide (https://cat-map.wustl.edu) making these promising candidate genes for mutation screening after the crystallins. In connexin 50, nearly 50% of identified mutations linked with different types of congenital cataract have been identified in the EL1 loop (e.g., V44M, V44E, V44A, W45S, W45R, W45L, G46V, G46R, D47N, D47H, D47Y, E48K, D51N, F52L, T56P, P59A, G60S, G60R, V64G, D67G, A69T, F70L, S73P, S73F, R76G, R76C, and R76H) and EL2 loop (P189A, P189L, V196M, R198W, R198Q, P199S, E201K, and T203NfsX47), in multiple families especially ADCC, indicating this region to be critical for the proper functioning of the gap junctions. In the present study, both threonine 56 and tryptophan 45 are located within the EL1 in connexin 50 and connexin 46 (Figures 1F, 2F, respectively), and both these residues are highly conserved in different species (Figures 1D, 2D, respectively) and in different human connexins (Figures 1E, 2E, respectively). EL1 and EL2, which are structurally essential for the formation of gap junctions, in mediating hemichannel docking are also instrumental in regulating the voltage gating of the channel. Therefore, any sequence change may potentially interfere with their conformation and voltage gating. Beyer et al. (19) for p.G46V substitution in connexin 50 predicted that mutants, especially in this region, would affect function if the mutant connexin was appropriately targeted to the plasma membrane.

Hadrami et al. (25) analyzed a nine-generation Mauritanian family living in an isolated village in the south of Mauritania, using a custom-designed in-solution capture array targeting a panel of 116 cataract candidate genes. Interestingly, for congenital nuclear cataract in that Mauritanian family, the authors identified a novel substitution, that is, c.166A > G (p.Thr56Pro), in *GJA8* involving the substitution of identical threonine at codon 56, however, with a different amino acid, that is, proline, as compared with our analyzed ADCC family (PC-04) having Thr56 replaced by alanine and was linked with a different phenotype, that is, total cataract. The authors further hypothesized that p.Thr56Pro mutation is ethnically specific. By bioinformatics analysis, p.Thr56Pro substitution was predicted to result in the disruption of *β*-sheet in connexin 50 and its replacement by a turn and a coil in the mutant protein, and this conformational change was assessed functionally as probably damaging with a score of 1.0 by SIFT and PolyPhen2, respectively. In the present Indian ADCC family (PC-04), SIFT and PolyPhen2 predicted the c.166A > G (p.Thr56Ala) variant in *GJA8* as damaging (with a score of 0.002 and 0.998, respectively). PROVEAN also predicted this change as damaging, indicating a score of −4.95 (Supplementary Table S2). HOPE predicted that despite the smaller size of the mutant Ala56, it is more hydrophobic than the wild-type Thr56 and hence may result in loss of hydrogen bonds and/or disturbed connexin 50 folding. Several clusters of water molecules that are buried at different sites in EL1/2 form hydrogen bonds with the peptide backbone and contribute to the architectural integrity of EL1/2 docking domains (23). Replacement of proline by more hydrophobic alanine might disturb this structural integrity and hence can affect the hemichannel docking. Also, threonine at codon 56 lies in the conserved docking HB-forming residues (54–57); hence, a point mutation at T56 might disturb the hemichannel docking.

In connexin 46, approximately 20 mutations linked with different cataract phenotypes are detected in the EL1 and EL2 loops, which are reported to be essential for docking interactions between hemichannels (26, 27). In another ADCC (PC-12) family with nuclear cataract in the present study, we have detected p.Trp45Ser substitution in connexin 46. p.Trp45Ser is also predicted to be damaging by SIFT (score 0.00), PolyPhen2 (score 1.0), and PROVEAN (score −13.41) (Supplementary Table S3). Additionally, HOPE predicted mutant Ser45 residue to be smaller than the wild-type residue and the wild-type residue to be more hydrophobic than the mutant residue. Due to the smaller size of the mutant Ser45, hydrophobic interactions might get lost either in the core of the connexin 46 or on the surface of the protein. p.Trp45Ser mutation has previously been linked with a similar phenotype, that is, nuclear cataract, in a Chinese ADCC family with 12 affected members in four generations (28). The authors further reported that the mutant proteins with p.Trp45Ser may disrupt normal interactions between the two connexons, which may reduce the resistance of the intercellular channel to the leakage of small ions. Rubin et al. (29) studied the voltage-dependent gating difference between Cx26 and Cx32 and reported that although EL1 and EL2 are instrumental in docking between connexons, and EL1 has a significant role in determining the trans-junctional voltage required for the closure of gap junction pores, any such defect in gap junction channels' structure and functioning seem to be detrimental in maintaining eye lens transparency. Zhang et al. (30) for the identical substitution, that is, c.134G > C (p.Trp45Ser) using *in silico* analysis tools I-Mutant, Mupro, and INPS-MD predicted that mutation might disturb the structure and function of the protein. Mutations in *GJA3* have been frequently reported with nuclear-type cataract in both humans and mice (24, 31). Interestingly, identical substitution, that is, c.134G > C (p.Trp45Ser) however, in *GJA8* as well has previously been reported by us in a three-generation ADCC family from India with five affected members having “jellyfish-like” cataract in association with microcornea (32) and in an another ADCC family from China with nuclear-type cataract (28). Apart from p.Trp45Ser substitution, the Trp45 in *GJA8* is reported to be substituted with other residues as well, that is, p.Trp45Arg in an ADCC family (with nuclear progressive cataract, microcornea, microphthalmia) from China (33), and p.Trp45Leu in three ADCC families: one from Iran with bilateral congenital cataract (in association microphthalmia and nystagmus) (34), another ADCC family from the United Kingdom with zonular pulverulent cataract (35), and a third family with dense congenital cataract (region not specified) (36). This indicates an essential role for tryptophan-45 in Cx46 as well as in Cx50 in maintaining eye lens transparency.

Diverse mechanisms for connexin 46 and connexin 50 mutants have been elucidated for cataractogenesis. These include variation in intercellular communication due to channel alterations or impaired cellular trafficking leading to the reduced number of effective gap junction channels within the plasma membrane/generation of nonfunctional intercellular channels/formation of hemichannels with altered gating or charge selectivity properties/gain of hemichannel function resulting in cell injury and death/formation of cytoplasmic accumulations and alterations in lens size, especially in case of connexin 50 protein (in mice cell line).

In summary, we describe a novel missense mutation c.166A > G (p.Thr56Ala) in *GJA8* linked with bilateral congenital total cataract in three members in a two-generation family from North India. p.Thr56 in *GJA8* seems to be a mutation hotspot as previously seen in an ADCC Mauritanian family, however, with a different phenotype, that is, nuclear cataract has been reported to harbor a different substitution, that is, p.Thr56Pro at the same codon. In another ADCC family with 11 members in four generations having nuclear cataract, c.134G > C (p.Trp45Ser) mutation in *GJA3* has been detected. Trp45 in *GJA3* as well as in *GJA8* seems to be mutation hotspots as the identical substitution in *GJA3* has previously been reported with similar phenotype in an ADCC family from China. Similarly, p.Trp45Ser, p.Trp45Arg, and p.Trp45Leu in GJA8 are reported to be linked with different cataract phenotypes inherited in autosomal dominant mode in families of different origins. As per the patient’s postoperative cataract extraction history, in cases with congenital cataract, early diagnosis and treatment are very important in terms of visual prognosis and avoiding secondary complications. Mackay et al. (37) documented that the timing of cataract extraction in patients with bilateral congenital cataract is critical to prevent amblyopia and they recommended that the age of cataract extraction can be from 10 weeks to a maximum of 1 year. In addition, identifying the molecular basis of congenital cataract is equally essential to providing individualized genetic counseling, prenatal diagnosis, and possibly preimplantation genetic diagnosis in the future.

## Data Availability

All relevant data is contained within the article: The original contributions presented in the study are included in the article/supplementary material. Further inquiries can be directed to the corresponding author.
