# Supplementary material for: Molecular diagnosis of autosomal dominant congenital cataract in two families from North India reveals a novel and a known variant in GJA8 and GJA3
Source: Front Pediatr. 2022 Dec 2;10:1003909. doi: 10.3389/fped.2022.1003909 (PMC9755747; doi:10.3389/fped.2022.1003909)
Supplement: Supplementary file 1 [file Table1.docx]

**Supplementary Table 1. Primer details of the candidate genes screened in the present study**

|  | **Forward primer (5' to 3')** | **Reverse primer (5' to 3')** | **Amplicon size (bp)** |
| --- | --- | --- | --- |
| ***CRYαA*** |  |  |  |
| EXON1 | GCCACGTTTCTGCTGACTG | CACTTCATCCCCTTGCAGAG | 505 |
| EXON2 | AGAAGAGGCCGGCAGGTGAC | CCTTCAACCCTGGGAGAG | 361 |
| EXON3 | CGCCAGGGTGCAAAATGC | TGGCCCTGAGGGTTTGGAGA | 370 |
| ***CRYαB*** |  |  |  |
| EXON1 | NON-CODING |  |  |
| EXON2 | TGCATATATAAGGGGCTGGC | CTGGAACCTCCTGGAAACTTC | 337 |
| EXON3 | TGCAGAATAAGACAGCACCTG | GTGCTATCAGCTTTGGAGGC | 286 |
| EXON4 | AGGATGCCTGAGTTCTGGG | CCCCACCAGTGAATGAAAGT | 343 |
| ***CRYβA1/A3*** |  |  |  |
| EXON1 | GAGCAGAGTGCTGACAGGG | CTCCTTGCCCTGCAGAGAC | 162 |
| EXON2 | CGTGTGTGCTCTGTCTTCC | CCATAAGGCAGTGACTGACC | 181 |
| EXON3 | CAATCCTCCCTCCACCTC | CCAAAGCTAGAAGGAAGG | 520 |
| EXON4 | CAAACACTACATGTCTTTGG | GCATATGAGGGATGGGAC | 210 |
| EXON5 | TGCTTCCTTGTATAATCC | CCTGAGGTTGCATCAATAGT | 306 |
| EXON6 | AAAGAGGCTCAGGTTTTGGG | CTGCTCACAGACATTGCTTTC | 320 |
| ***CRYβA2*** |  |  |  |
| EXON1 | GCTGTGTGTGGCTCGAAAC | CACCGGCCTAGGCTCAG | 299 |
| EXON2 | GGAGGGCAGAGAGGGTG | CACCATGGCACCTAGAAAGG | 271 |
| EXON3 | TCCGTTAACCCTTTGGAAGC | CAAGGAAGAGGGTGGAGAAA | 272 |
| EXON4 | TGGTGGAGAACATAGGGTGG | CTGAATCTTCCTGCCCCTCT | 283 |
| ***CRYβA4*** |  |  |  |
| EXON1 | NON-CODING |  |  |
| EXON2 | AGCCAGCCATCTGCATAAAG | GGGGAGGGTTCAGGTCC | 177 |
| EXON3 | CCCCAACCTCTCACCCTTC | GGACAGGAAGGGACCTAGAGA | 241 |
| EXON4 | TTGCTGGTCTAGAATGCAGG | CTTCGGAATCAAAGGTTCCA | 281 |
|  |  |  |  |
| EXON5 | GCTCCTGGGTTTCCAACTG | GGGAGGGGTAGGTGTACCTC | 262 |
| EXON6 | CCCTGTGTTGATCACCATGC | CTGGAGGCTGTGGTGTGTT | 283 |
| ***CRYβB1*** |  |  |  |
| EXON1 | NON-CODING |  |  |
| EXON2 | GAGAGGAAACGAGCTCCAAG | CCTCTTACTCCTCCGCACCT | 414 |
| EXON3 | GCACTGCTGGCTTTTATTTATG | CACACAACAGTAGCTGCCATT | 278 |
| EXON4 | AAAGGCAAGTAGGGAGTGGG | CAGGGCAGGTGTAAGTCTGAG | 272 |
| EXON5 | GCCCCGCTAAGTTTCTTCTC | CACAGGCAGAATCAGAGGCT | 273 |
| EXON6 | GCCAGGCAACGAGACAG | GTTACTGGGATGTGACTTCCTC | 401 |
| ***CRYβB2*** |  |  |  |
| EXON1 | NON-CODING |  |  |
| EXON2 | GTTTGGGGCCAGAGGGGAGTGGT | TGGGCTGGGGAGGGACTTTCAGTA | 350 |
| EXON3 | TCAGCATCCTTTGGGTTCTC | AGTGGGGGAATCTACCCTTG | 299 |
| EXON4 | AACCCTAGGGGTCAACATCA | CTCTCTCTGCCACCTTGGAG | 268 |
| EXON5 | GAGTGATGTGTGGGACATGC | GTGTGCTCTGCTGACCTCTG | 377 |
| EXON6 | CTTACCCTTGGGAAGTGGCAATGG | TCAAAGACCCACAGCAGACAAGTT | 600 |
| ***CRYβB3*** |  |  |  |
| EXON1 | NON-CODING |  |  |
| EXON2 | CCAGTCACATCAACACCTGG | GGTCATCTTCCCAGCCAAG | 229 |
| EXON3 | CTCTAATGCCCAAAGGAGGG | CCTTCTGGGAGTGTGGAGG | 257 |
| EXON4 | AAACTTGAATCCTTCCTCAGC | GAAGCAGGGTGCACTGAGA | 249 |
| EXON5 | AGCCTCCTTGACCTCTGTTC | AACAGAATCAGTGCCCATAGT | 285 |
| EXON6 | AAGAGGAATGTAGGCAGGCAGAGT | AATAAAGCCTGGGGTTGGTCC | 401 |
| ***CRYγA*** |  |  |  |
| EXON1-2 | ATACCAGTTGCCCCTTTGTC | GTCAGTTTGTTCCAACACAGGA | 505 |
| EXON3 | CACTTTCGTTGACACCCAAG | GTTCCCTGCACTAAGTGGCT | 412 |
| ***CRYγB*** |  |  |  |
| EXON1-2 | TGCAAATCCCCTTACTCACC | GCATCCGGCCTATTTAATCA | 861 |
| EXON3 | AGAACTTCTGGGCAGGTGTG | GAGCCCTGTCTTTCTTTCAA | 469 |
| ***CRYγC*** |  |  |  |
| EXON1-2 | CCTGCTATATAGACTGGCTGTGC | GTCAATGTGGGTTACAGGGAG | 492 |
| EXON3 | CTTTGGTTGGACAAATTCTGG | CTGTCATTCTTTCATTGTGCAA | 440 |
| ***CRYγD*** |  |  |  |
| EXON1-2 | GCAGCCCACCCGCTCA | CTCCCCACATAAGCAAAGTATT | 492 |
| EXON3 | TGCATTGGAATCATTTCACAC | CTGGCACTGATTTGCTTGTG | 410 |
| ***CRYγS*** |  |  |  |
| EXON1 | NON-CODING |  |  |
| EXON2 | ATGTTTGACTGAAACCAGCC | GCTTTTGCTTCTTTGCCTAGA | 214 |
| EXON3 | TGTGAATTAAGCCACCCAGC | CGCTTTCTCTTGCTTTTAGGTG | 367 |
| EXON4 | TTGACCTGCTGGTGATTTCC | CTGGCCTTGTGGTCCAAATA | 394 |
| ***GJA3*** |  |  |  |
| EXON2_1 | ATGCCTGTCCTGTGGAGAAG | CGTCCCTGCTGCTCAAC | 802 |
| EXON2_2 | CTGAAGAGAGAGAGCCCCAG | CAGTCCGCCAAGCTCTACA | 604 |
| EXON2_3 | GGGTTCCCACCCTACTATGC | GCACTTCCCACCAGCAGATA | 594 |
| ***GJA8*** |  |  |  |
| EXON2_1 | TCTGCACAAAGGAAGCACTG | CTGTGTCCCTATTCCTCAACG | 824 |
| EXON2_2 | AGGGCAGCGTCAAGAAGAG | GTTCGAGGAGAAGATCAGCA | 540 |
| EXON2_3 | TCGTTTCCCACTATTTCCCC | CGTAGGGCAAGATGAAAGGA | 540 |
