# Supplementary material for: Molecular diagnosis of autosomal dominant congenital cataract in two families from North India reveals a novel and a known variant in GJA8 and GJA3
Source: Front Pediatr. 2022 Dec 2;10:1003909. doi: 10.3389/fped.2022.1003909 (PMC9755747; doi:10.3389/fped.2022.1003909)
Supplement: Supplementary file 2 [file Table2.docx]

**Supplementary Table 2. Functional prediction results of c.166A>G (p.Thr56Ala) in *GJA8* using different softwares and arithmetics**

| **Algorithm** | **Score** | **Prediction** | **Damaging score criteria** |
| --- | --- | --- | --- |
| SIFT | 0.002 | Damaging | ≤0.05 damaging; >0.05 tolerable |
| Polyphen-2_HDIV | 1 | Probably_damaging | ≥0.453 probably damaging; <0.453 tolerable |
| Polyphen-2_HVAR | 0.998 | Probably_damaging | ≥0.447 probably damaging; <0.447 tolerable |
| LRT | 0 | Deleterious | ≤0.001 deleterious; >0.001 tolerable |
| MutationTaster | 1 | Disease_causing | >0.5 disease causing; ≤0.5 tolerable |
| MutationAssessor | 3.58 | Damaging | >1.9 damaging; ≤1.9 tolerable |
| FATHMM | -5.74 | Damaging | ≤1.5 damaging; >1.5 tolerable |
| PROVEAN | -4.95 | Damaging | ≤2.5 damaging; >-2.5 tolerable |
| VEST3 | 0.91 | Damaging | ≥0.5 damaging; <0.5 tolerable |
| MetaSVM | 1.012 | Damaging | >0 damaging; ≤0 tolerable |
| MetaLR | 0.986 | Damaging | >0.5 damaging; ≤0.5 tolerable |
| M-CAP | 0.872 | Damaging | >0.025 damaging; ≤0.025 tolerable |
| CADD | 24.6 | Damaging | >20 damaging; ≤20 tolerable |
| DANN | 0.997 | Damaging | ≥0.99 damaging; <0.99 tolerable |
| FATHMM_MKL | 0.965 | Damaging | >0.5 damaging; ≤0.5 tolerable |
| Eigen | 0.853 | Damaging | ≥0 damaging; <0 tolerable |
| GenoCanyon | 1 | Damaging | >0.999 damaging; ≤0.999 tolerable |
| fitCons | 0.497 | Tolerable | >0.7 damaging; ≤0.7 tolerable |
| GERP++ | 5 | Conserved | ≥2 DNA sequence is conserved |
| phyloP | 9.249 | Conserved | >2 DNA sequence is conserved |
| phastCons | 1 | Conserved | >0.999 DNA sequence is conserved |
| SiPhy | 14.674 | Conserved | ≥12 DNA sequence is conserved |
| REVEL | 0.979 | Damaging | ≥0.4 is damaging; <0.4 tolerable |
| ReVe | 0.978 | Damaging | ≥0.4 is damaging; <0.4 tolerable |
| ClinPred | 0.995 | Pathogenic | ≥0.5 is damaging; <0.5 tolerable |
